# CCL7-CCR3 signaling mediates olfactory dysfunction in a mouse model of allergic rhinitis

**DOI:** 10.3389/fimmu.2026.1893377

**Published:** 2026-08-10

**Authors:** Ting Yang, Hui Shen, Tingting Zhao, Yakui Mou, Yue Hu, Xiaoyu Song, Yao Wang, Yujiao Chen, Hanrui Wang, Chao Ren, Xicheng Song

**Affiliations:** 1 Shandong Provincial Clinical Research Center for Otorhinolaryngologic Diseases, Yantai Yuhuangding Hospital, Qingdao University, Yantai, China; 2 Shandong Provincial Key Laboratory of Neuroimmune Interaction and Regulation, Yantai Yuhuangding Hospital, Qingdao University, Yantai, China; 3 Department of Otorhinolaryngology, Head and Neck Surgery, Yantai Yuhuangding Hospital, Qingdao University, Yantai, China; 4 Shandong Engineering Research Center for Precision Diagnosis and Treatment of Airway Diseases, Yantai Yuhuangding Hospital, Qingdao University, Yantai, China; 5 Yantai Key Laboratory of Otorhinolaryngologic Diseases, Yantai Yuhuangding Hospital, Qingdao University, Yantai, China; 6 Yantai Clinical Research Center for Otorhinolaryngologic Diseases, Yantai Yuhuangding Hospital, Qingdao University, Yantai, China; 7 Department of Neurology, Yantai Yuhuangding Hospital, Qingdao University, Yantai, China; 8 Yantai Municipal Key Medical and Health Laboratory of Yantai yuhuangding hospital (Interdisciplinary Brain Science and Geriatric Health Laboratory), Yuhuangding Hospital, Qingdao University, Yantai, China

**Keywords:** allergic rhinitis, C-C motif chemokine ligand 7 (CCL7), microglia, olfactory bulb, olfactory dysfunction

## Abstract

**Background:**

Allergic rhinitis (AR) is a prevalent chronic upper airway inflammatory disorder. Olfactory dysfunction (OD) in AR patients represents a frequent and burdensome complication that significantly compromises quality of life. While the pathogenesis of AR-associated OD remains incompletely characterized, emerging evidence points to olfactory bulb (OB) microglial neuroinflammation as a crucial contributor. C-C motif chemokine ligand 7 (CCL7) is consistently upregulated in AR nasal mucosa and has been documented to drive microglial inflammation. However, its expression and function in OB microglia remain undefined. This study aimed to investigate the specific role of CCL7 in OB microglia and its potential contribution to AR-associated OD, with the goal of providing new mechanistic insights and potential intervention targets.

**Methods:**

An ovalbumin (OVA)-induced murine AR-associated OD model was established and validated through behavioral, serological, and histopathological analyses. CCL7 and C-C chemokine receptor type 3 (CCR3) expression profiles in OB and nasal mucosa were evaluated by qPCR, ELISA, and immunofluorescence colocalization with microglial markers. The therapeutic efficacy of intranasal CCL7 inhibition was assessed using Bindarit over a sustained intervention period. The specificity of the CCL7-CCR3 axis was further validated by intranasal exogenous CCL7 administration in naive mice and stereotaxic injection of the CCR3-specific antagonist SB-328437 into the OB of AR mice with established OD. *In vitro* studies employed co-culture with allergen-stimulated nasal epithelial and microglial cells, with or without CCR3 antagonist-pretreatment.

**Results:**

CCL7 expression was significantly elevated in OB of AR-associated OD mice, correlating positively with behavioral indices of OD. CCR3 was the only significantly upregulated CCL7 receptor in OB in this model, selectively localized to activated microglia. Intranasal Bindarit progressively improved olfactory function in AR mice, with marked improvement first observed at week 8 of treatment, with concomitant reduced CCL7 and neuroinflammatory mediators in OB. In contrast, intranasal exogenous CCL7 administration in naive mice recapitulated OD and upregulated these neuroinflammatory markers. Conversely, CCR3-specific antagonism with SB-328437 in the OB partially restored olfactory function in AR mice with established OD. *In vitro*, allergen-stimulated nasal epithelial cells secreted CCL7 that activated microglial cells through CCR3, an effect partially reversed by CCR3 antagonist.

**Conclusion:**

The CCL7-CCR3 signaling axis links nasal allergic inflammation to OB microglial neuroinflammation and consequent OD in AR, identifying a promising therapeutic target for AR-associated OD.

## 1 Introduction

Allergic rhinitis (AR) is a highly prevalent chronic inflammatory disorder of the upper airway, affecting approximately 10−40% of the global population (1). Among its diverse sequelae, olfactory dysfunction (OD) represents a particularly burdensome complication, occurring in up to 42.9% of patients with AR and rising to 88% in those with comorbid chronic rhinosinusitis and nasal polyps (2). This impairment substantially compromises patient quality of life, with affected individuals frequently developing anxiety, depression, cognitive deficits, and sleep disturbances that further amplify the socioeconomic burden (3, 4). Despite increasing clinical attention, the pathogenesis of AR-associated OD remains unclear, and therapeutic strategies targeting its prevention and reversal remain limited. This reflects an incomplete understanding of the underlying pathophysiological mechanisms and underscores the urgent need to identify specific, actionable intervention targets.

Chemokine research has garnered increasing attention in the pathophysiology of allergic diseases (5). Chemokines orchestrate immune cell trafficking through G protein-coupled receptor interactions (5), with their roles extensively documented in AR, asthma, and atopic dermatitis (6–12). In AR specifically, nasal mucosal profiling revealed upregulation of multiple CC chemokines, including C-C motif chemokine ligand (CCL)1, CCL2, CCL5, CCL7, CCL8, CCL11, CCL13, CCL17, and CCL24, together with their cognate receptors C-C chemokine receptor type (CCR)2, CCR3, CCR4, CCR5, and CCR8 (13). CCL7, also designated monocyte chemoattractant protein-3 (MCP-3), is a versatile CC chemokine that exhibits pleiotropic binding to CCR1, CCR2, and CCR3 (14). It orchestrates recruitment and activation of eosinophils, monocytes, and T lymphocytes, and is associated with allergic and inflammatory disease pathologies (15–23). CCL7 is consistently elevated across multiple biological compartments in AR, with nasal mucosal mRNA upregulation exceeding 90% compared with healthy controls (13), and demonstrates increased protein secretion following allergen challenge (24). Furthermore, Zhang et al. demonstrated that the CCL7-CCR3 axis specifically mediates eosinophil chemotaxis in AR (25). Notably, CCL7 drives microglial inflammation in retinitis pigmentosa and is a key regulator of microglial function (26). We previously demonstrated that OB microglial neuroinflammation represents a pivotal mechanism underlying AR-associated OD (27, 28). However, whether CCL7, a chemokine consistently elevated in AR peripheral compartments, similarly mediates microglial activation within OB and contributes to AR-associated OD pathogenesis remains unclear. Given that CCL7 is elevated in the nasal mucosa and nasal secretions of patients with AR as a pathogenic inflammatory factor, and considering that OB neuroinflammation may originate from nasal mucosal inflammatory signal transmission, we hypothesized that CCL7 is expressed in OB microglia and serves as an important mediator of AR-associated OD. The present study was designed to test this hypothesis and elucidate the specific role of CCL7 in linking nasal allergic inflammation to OB microglia and consequent OD in AR. We established animal and cellular models of AR to demonstrate that CCL7-CCR3 signaling mediates OB microglial activation and identified a novel potential therapeutic target for AR-associated OD.

## 2 Materials and methods

### 2.1 Animals

The animal research protocol was approved by the Ethics Committee of Yantai Yuhuangding Hospital (Approval No. 2024-573). C57BL/6 male mice, aged 6−8 weeks and weighing 22−25 g, were procured from GemPharmatech (Nanjing, China). All mice were housed in a temperature-controlled room (22−24 °C) on a 12-h light/dark cycle (from 7:00 am to 7:00 pm) with ad libitum access to food and water. The animals were housed and cared for according to the Guidelines for the Care and Use of Laboratory Animals (ISBN: 9787547812969). All experimental procedures were conducted in compliance with the ARRIVE guidelines and in accordance with the U.K. Animals (Scientific Procedures) Act 1986 and associated guidelines (EU Directive 2010/63/EU).

### 2.2 Establishment of a murine AR model

The murine AR model was established using a method described previously (28). On Days 1−14, the AR group was sensitized by intraperitoneal injection of 200 μL normal saline containing 40 μg ovalbumin (OVA; A8040, Solarbio, Beijing, China) and 2 mg aluminum hydroxide (OZB-AH0050, OZ Biosciences, France) every 2 days. The control group was injected with the same amount of pure normal saline. The challenge phase was initiated from Day 21. The AR group was administered 5% OVA (diluted with normal saline) via nebulization for 30 min, followed by intranasal administration of 20 μL per nostril once daily for seven consecutive days. The control group underwent the same procedures with an equivalent volume of pure normal saline. Beginning on Day 28, the maintenance phase was initiated. Mice in the AR group received intranasal administration of 5% OVA every other day, combined with a weekly 30-min nebulization of 5% OVA. Control mice received intranasal and nebulized administration of sterile normal saline in equivalent volumes.

### 2.3 Pharmacological interventions

To evaluate the therapeutic potential of CCL7 inhibition, mice in the CCL7 inhibitor-treated group received 5 mg/kg of CCL7 inhibitor (Bindarit, HY-B0498, MCE, Shanghai, China) intranasally every other day from the maintenance phase of AR model establishment. Administration was scheduled 30 min post each 5% OVA nasal challenge and continued for 16 weeks, with concurrent weekly behavioral testing performed to monitor olfactory performance alterations throughout the study duration. In a separate cohort of naive mice, the CCL7-treated group received intranasal administration of 100 ng CCL7 (rmCCL7, 250-08, PeproTech, USA) once daily for the first week and every other day for the second week, whereas the control group received an equal volume of PBS following the same schedule. Behavioral tests were initiated on day 14. For CCR3-specific antagonism, SB-328437 (100 ng per side, HY-103363, MCE, Shanghai, China) or an equal volume of vehicle was injected bilaterally into the OB of AR mice with established OD via stereotaxic surgery. Mice were anesthetized with isoflurane and secured in a stereotaxic frame. A glass micropipette was lowered into the OB (AP: +4.13 mm, ML: ± 0.80 mm, DV: -1.00 mm relative to bregma), and the drug or vehicle was infused bilaterally at 30 nL/min. The pipette was left in place for 10 min post-injection before slow withdrawal, followed by wound closure and recovery on a warming pad. Behavioral tests were performed 24 h after the injection.

### 2.4 Animal behavior experiments

Following instillation of 5% OVA during the final intranasal allergen challenge in the model establishment period, the mice were observed for 30 min, and their scratching responses were quantified. A 3-min interval was randomly selected from each group, during which the number of nasal scratching events was recorded. For the buried food pellet test (BFPT), a 5 cm layer of bedding was placed in the experimental box (40 cm × 24 cm × 20 cm), ensuring that the density of the bedding was as uniform as possible. Three days prior to the start of the experiment, the mice were placed in the box for approximately 30 min to familiarize them with the test environment. The mice were then fasted for 12 h. Food pellets (0.5 g) were randomly buried in the bedding at a depth of about 0.5 cm. The latency to uncover the food pellet, defined as grabbing a pellet with the forepaws, was recorded, and the average of three measurements was calculated. Mice failing to locate a pellet within 300 s were recorded as exhibiting OD. Fresh bedding was used for each test (29).

### 2.5 Sample collection

After completion of the behavioral tests, mice were anesthetized with 2% isoflurane. To ensure painless collection, blood was drawn from the retro-orbital sinus in 5 mL blood collection tubes. The nasal mucosa was harvested following sacrifice. The skin tissue of the mouse head was bluntly dissected under a microscope to expose the skull. The junction of the zygomatic bone and the maxilla on both sides of the skull was cut with scissors. The nasal bone above the nostril was pried with tweezers, which were also used to separate the soft tissue covering the roof of the palate, exposing the floor of the nasal cavity. Furthermore, the maxillary alveolar bone was then truncated with tweezers, and the bone at the upper teeth and the tip of the nose was excised with scissors. At the base of the maxilla, the bony septum was disconnected from the bone of the left and right maxilla using a blade or scissors. Tweezers were used to clamp the anterior maxilla and pry the maxillary bone to both sides. The nasal septum was clamped at its base and removed with tweezers cautiously so as to preserve the integrity of the nasal mucosa. The nasal mucosa covering the left and right maxilla was then visualized. Using needle forceps, the mucosa covering both sides of the bony nasal septum was removed and preserved. To collect the OB, which is located at the front end of the telencephalon and connected with olfactory filaments, the parietal bone was removed. Brain tissues and OB were carefully dissected, and the OB tissue was separated. All tissue samples were stored in a -80 °C freezer until further testing.

### 2.6 Cell culture and treatment

Human nasal epithelial (HNEpC) and human microglial clone 3 (HMC3) cell lines were purchased from the BeNa Co., Ltd. (Beijing, China). The cells were cultured in minimum essential medium (MEM; C3050, BioInd, Israel) supplemented with 10% fetal bovine serum (FBS; C04001, BioInd, Israel) and 1% penicillin-streptomycin solution (C0222, Beyotime, Beijing, China) at 37 °C with 5% CO_2_. The dermatophagoides pteronyssinus 1 (Der p 1; XPB91D3A2.5, Greer, USA) was dissolved in sterile injection water. Once the HNEpC cell density reached 70−80%, the cells were treated with 40 µg/mL Der p 1 for 24 h. The supernatant was then collected to prepare conditioned medium (CM). After the HMC3 cells reached 60−70% confluence, the CCR3-specific inhibitor SB-328437 (HY-103363, MCE, Shanghai, China), dissolved in dimethyl sulfoxide (DMSO; 67-68-5, MP Biomedicals, USA), was pre-applied for 1 h. Subsequently, CM and normal culture medium were mixed at a 1:1 ratio and co-cultured with HMC3 cells for 24 h.

### 2.7 Enzyme-linked immunosorbent assay

Mouse blood samples were collected and allowed to stand for 2−4 h. The samples were then centrifuged at 3,000 rpm for 10 min at 4 °C, and the resulting serum was used to measure immunoglobulin E (IgE), interleukin (IL)-4, and IL-5 levels. The serum levels of IgE, IL-4 and IL-5 were quantified using the corresponding ELISA kits (NeoBioScience, China) in accordance with the manufacturer’s instructions.

OB and nasal mucosa were collected and rinsed with pre-cooled phosphate buffered saline (PBS; pH 7.4) to eliminate residual blood. The tissues were minced and transferred into grinding tubes containing 200−250 μL PBS supplemented with 1 μL phenylmethylsulfonylfluoride (PMSF; EA0005, SparkJade, China). The tissues were homogenized using 2 mm stainless steel beads in a Tissuelyser (Shanghai Jing Xin, China) at 60 Hz for 300 s. The lysates were then incubated on ice for 25 min and centrifuged at 13,000 rpm for 15 min at 4 °C. The supernatants were collected and stored for subsequent assays. CCL7 and CCR3 protein levels in the lysates were then measured using mouse CCL7 (JL12836, Jonlnbio, China) and mouse CCR3 (JL31120, Jonlnbio, China) ELISA kits.

For the *in vitro* experiments, cell culture media were collected from Der p 1-treated and control groups and centrifuged at 1,000 rpm for 20 min at 4 °C. The clarified supernatants were then collected and CCL7 levels in the supernatants were analyzed using human CCL7 ELISA kit (JL11114, Jonlnbio, China) according to the manufacturer’s instructions.

Subsequent steps were performed in accordance with the kit instructions. A Multiskan FC zymograph (Thermo Fisher Scientific, USA) was used to measure the absorbance at 450 nm and the protein concentration was calculated.

### 2.8 Quantitative real-time polymerase chain reaction

Total RNA was extracted from tissues and cell lines using TRIzol^®^ reagent (AC0101, SparkJade, China) according to the manufacturer’s instructions. The RNA concentration was measured, and cDNA was synthesized by reverse transcription using the First-Strand cDNA Synthesis Kit (AG0302; SparkJade, China). Then, qPCR was performed using the 2× ChamQ SYBR^®^ qPCR Master Mix (Q311-02, Vazyme, Jiangsu, China) on the FTC-3000P system (Funglyn Biotech, Canada). The thermal cycling program was set as follows: initial denaturation at 95 °C for 5 min; 40 cycles of denaturation at 95 °C for 10 s and annealing/extension at 60 °C for 30 s; followed by 95 °C for 1 min, 60 °C for 1 min, 95 °C for 0.5 s. The data obtained were analyzed using the ΔΔCT method. Relative gene expression was calculated using the 2^-ΔΔCT^ cycling threshold method, using glyceraldehyde 3-phosphate dehydrogenase (GAPDH) as the internal control to calculate relative RNA expression levels. ΔΔCT = (CT_Target gene_ - CT_GAPDH_)_Time X_ - (CT_Target gene_ - CT_GAPDH_)_Time 0_, where Time X represents any time point, and Time 0 represents the baseline (defined as 1× expression of the target gene normalized to GAPDH). The primers (Sangon Biotech, Shanghai, China) used for qPCR are listed in **Table 1**. All samples were run in triplicate, and three independent biological replicates were analyzed.

**Table 1:** Primers used for qPCR in this study.

Species	Gene	Primer sequences (5’ to 3’)
Mouse	GAPDH	F:AGGTCGGTGTGAACGGATTTG
		R:TGTAGACCATGTAGTTGAGGTCA
	CCL2	F:CACTCACCTGCTGCTACTCA
		R:GCTTGGTGACAAAAACTACAGC
	CCL3	F:TACAAGCAGCAGCGAGTACC
		R:CTTCCGGCTGTAGGAGAAGC
	CCL5	F:GCAGTCGTGTTTGTCACTCG
		R:AGAGCAAGCAATGACAGGGA
	CCL7	F:CGCTGCTTTCAGCATCCAAG
		R:CTTGAAGATAACAGCTTCCCAGG
	CCL11	F:TGCTCACGGTCACTTCCTTC
		R:CTTGAAGACTATGGCTTTCAGGGTG
	CCL12	F:GGAAGCTGTGATCTTCAGGAC
		R:GGGGAACTTCAGGGGGAAATA
	CCL20	F:CAGGCAGAAGCAAGCAACTAC
		R:AGCTTCATCGGCCATCTGTC
	CCL24	F:ATGGCGGGCTCTGCTACG
		R:AGGGGATGGTCACAGAATCTATGG
	CCL26	F:CGATTTGGGTCTCCTTGTTCTCC
		R:GAAATTAGGGCAGCAGGACATAGC
	CXCL2	F:CCCAGACAGAAGTCATAGCCAC
		R:TGGTTCTTCCGTTGAGGGAC
	CXCL9	F:TGTGGAGTTCGAGGAACCCT
		R:AGTCCGGATCTAGGCAGGTT
	CXCL10	F:GCCTCATCCTGCTGGGTCTG
		R:TTCCCTATGGCCCTCATTCTCAC
	CXCL16	F:CCAGATACCGCAGGGTACTT
		R:CCAGTTCCACACTCTTTGCG
	CCR1	F:GAAGAAGGTCAAAGCCGTGC
		R:GGCAATCACCTCAGTCACCT
	CCR2	F:CTCTGCAAACAGTGCCCAGTT
		R:CTCCCCAGTGGAAGGAGTGA
	CCR3	F:TTACCTGGCCTTGTACAGCG
		R:GGATAGCGAGGACTGCAGGA
	CCR5	F:TGCTGTGTTTGCTTTAAAAG
		R:ATGACAAGTAGAGGCAGGAT
	IL-1β	F:GCAACTGTTCCTGAACTCAACT
		R:ATCTTTTGGGGTCCGTCAACT
	IL-6	F:AAGTGCATCATCGTTGTTCATACA
		R:GAGGATACCACTCCCAACAGACC
	iNOS	F:CGAAACGCTTCACTTCCAA
		R:TGAGCCTATATTGCTGTGGCT
Human being	GAPDH	F:CTGGGCTACACTGAGCACC
		R:AAGTGGTCGTTGAGGGCAATG
	Claudin-1	F:GTCTTTGACTCCTTGCTGAATCTG
		R:CACCTCATCGTCTTCCAAGCAC
	JAM-1	F:GTGAAGTTGTCCTGTGCCTACTC
		R:ACCAGTTGGCAAGAAGGTCACC
	IL-4	F:GCTTCCCCCTCTGTTCTTCC
		R:CTGCTCTGTGAGGCTGTTCA
	IL-5	F:TCCCACAAGTGCATTGGTGA
		R:CAGTACCCCCTTGCACAGTT
	IL-1β	F:AGCTACGAATCTCCGACCAC
		R:CGTTATCCCATGTGTCGAAGAA
	IL-6	F:ACTCACCTCTTCAGAACGAATTG
		R:CCATCTTTGGAAGGTTCAGGTTG
	CCL7	F:ATCCCTAAGCAGAGGCTGGA
		R:GTCCTGGACCCACTTCTGTG
	iNOS	F:GGCAAGCCCAAGGTCTATGT
		R:GGAGGCTCCGATCAATCCAG

qPCR, quantitative real-time polymerase chain reaction; GAPDH, glyceraldehyde 3-phosphate dehydrogenase; CCL, C-C motif chemokine ligand; CXCL, C-X-C motif chemokine ligand; CCR, C-C chemokine receptor; IL, interleukin; iNOS, inducible nitric oxide synthase; JAM, junctional adhesion molecule; F, Forward primer; R, Reverse primer.

### 2.9 Immunofluorescence staining of OB

According to the standard operation procedure, mice were deeply anesthetized with 2% isoflurane until loss of the pedal reflex. They were then euthanized by exsanguination during transcardial perfusion with physiological saline, followed by 4% paraformaldehyde (PFA; BL539A, Biosharp, China) to obtain complete snouts and OBs. Mouse OBs were fixed in 4% PFA at 4 °C overnight, and subsequently cryoprotected by sequential incubation in 20% sucrose for 24 h, followed by 30% sucrose until the tissue had fully sunk. Finally, the samples were embedded in optimal cutting temperature (OCT; 4583, SAKURA, USA) compound for cryo-sectioning. Serial 12 μm cryosections of OB tissues were obtained using a cryostat (FS800, RWD, China) and mounted on adhesive slides. The sections were air-dried, rinsed in PBS, and blocked with 5% bovine serum albumin (BSA; GC305010, Servicebio, China) in PBS for 1 h at room temperature. They were then incubated with rabbit anti-CCR3 (ab318266, 1:200, Abcam, Britain) primary antibodies at 4 °C overnight in a humidified box. The next day, the sections were washed three times in PBS (pH 7.4) for 5 min each. After brief drying, the sections were incubated with Alexa Fluor^®^ 594-conjugated goat anti-rabbit IgG (H+L) (GB28301, Servicebio, China) secondary antibody for 1 h at room temperature in the dark. The sections were washed three times in PBS (pH 7.4) for 5 min each. Next, serial sections were incubated separately with mouse anti-transmembrane protein 119 (TMEM119; #98778, 1:100, Cell Signaling Technology, USA) or mouse anti-ionized calcium-binding adapter molecule 1 (IBA1; ab283319, 1:200, Abcam, Britain) antibodies at 4 °C overnight for co-staining. After washing three times in PBS for 5 min each, the corresponding Alexa Fluor^®^ 488-conjugated goat anti-mouse IgG (H+L) (GB25301, Servicebio, China) secondary antibodies were added and incubated at room temperature for 1 h in the dark. After three additional PBS washes, the nuclei were counterstained with 4′,6-diamidino-2-phenylindole (DAPI; G1012, Servicebio, China) at room temperature for 10 min in the dark. The sections were then sealed with an anti-fluorescence quenching agent. Fluorescence images were acquired using an inverted fluorescence microscope (Axio Vert. A1, Zeiss, Germany) under identical exposure settings. Co-localization was quantitatively assessed by generating fluorescence intensity plot profiles across rectangular regions of interest using ImageJ software. For each channel, gray values were plotted against pixel distance, and co-localization was confirmed by the alignment of intensity peaks.

### 2.10 Histopathological evaluation of nasal mucosa

The snouts were fixed in 4% PFA (BL539A, Biosharp, China), decalcified in 10% EDTA decalcifying solution (E1171, Solarbio, China) at room temperature with regular replacement of the decalcification solution until complete decalcification, dehydrated in a graded ethanol series, and embedded in paraffin. Sections were sliced at 4 μm thickness and collected. The slices were stained with hematoxylin/eosin (H&E; C0105, Beyotime, Beijing, China) and Periodic Acid-Schiff (PAS; C0142M, Beyotime, Beijing, China) according to the manufacturer’s instructions. The processed tissue sections were subsequently visualized under a light microscope (DMLB2, Leica, Germany), and images were captured. To quantify eosinophil infiltration and goblet cell numbers, five randomly selected high-power fields per H&E-stained and PAS-stained section, respectively, were evaluated. The number of eosinophils in each field of H&E sections and the number of PAS-positive goblet cells in each field of PAS sections were counted. The mean cell count per field was calculated for each mouse. Representative images are shown with scale bars of 20 μm (for H&E) and 50 μm (for PAS).

### 2.11 Statistical analyses

All experiments were performed at least three times. The data are expressed as mean ± standard error of the mean (SEM). GraphPad Prism 9 software (San Diego, CA, USA) was used for statistical analysis of the experimental data. Two groups were compared using a t-test, and a one-way ANOVA was used for comparison among multiple groups. Statistical significance was set at *P* < 0.05.

## 3 Results

### 3.1 AR mice exhibit OD

**Figure 1:**
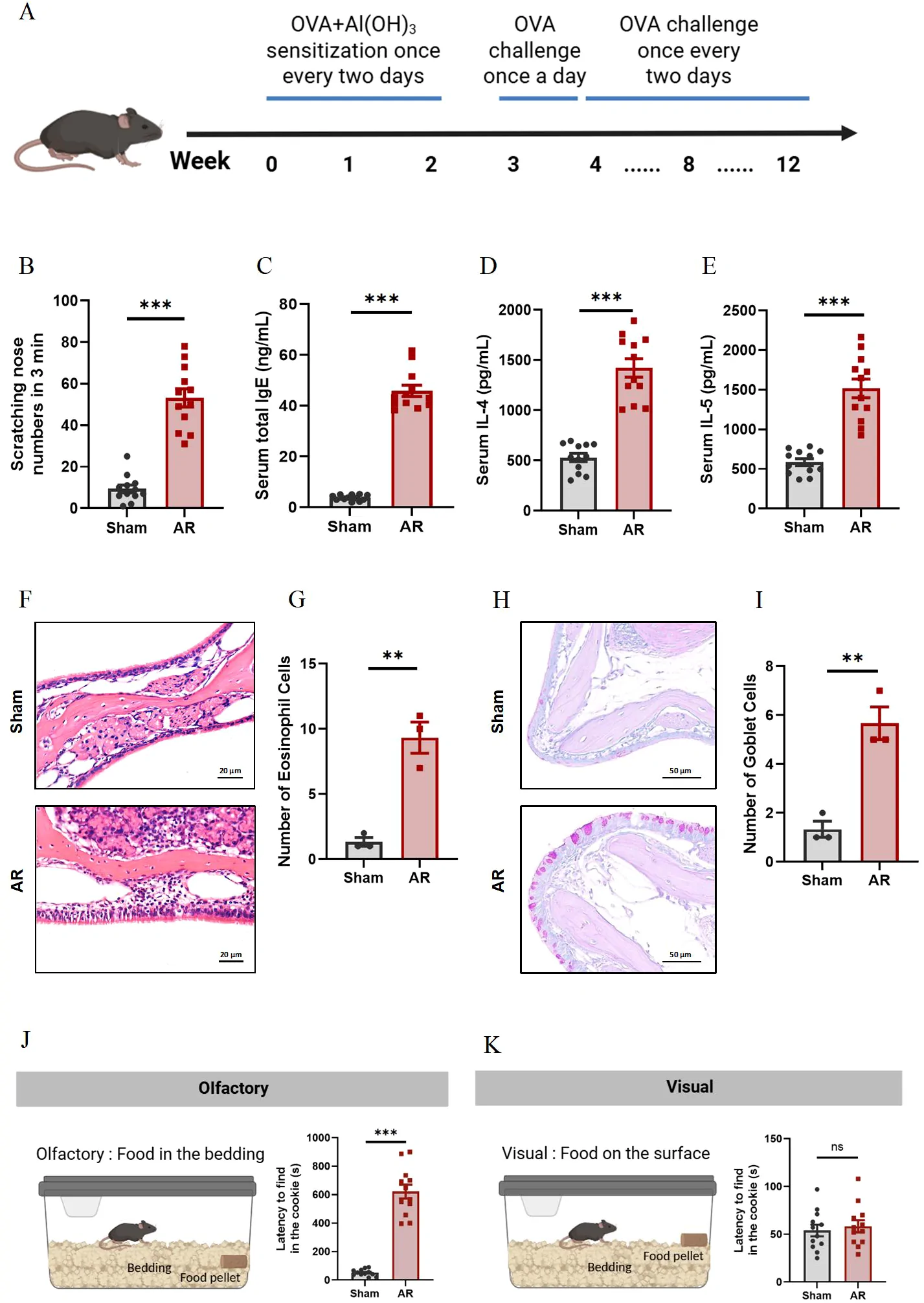
Evaluation of the ovalbumin (OVA)-induced allergic rhinitis (AR) mouse model and associated changes in olfactory function. **(A)** Schematic illustration of the experimental protocol and temporal schedule for AR mouse model establishment. **(B)** Statistical comparison of nasal scratching frequency in 3 min among different groups after the final intranasal allergen challenge. **(C)** Statistical comparison of serum total IgE levels between groups measured using enzyme-linked immunosorbent assay (ELISA). **(D)** Statistical comparison of serum total IL-4 levels between groups measured using ELISA. **(E)** Statistical comparison of serum total IL-5 levels between groups measured using ELISA. **(F)** Histomorphological changes in the nasal mucosa revealed by hematoxylin and eosin (H&E) staining in different groups. **(G)** The eosinophil count in nasal mucosa of mice between groups (scale bar = 20 μm). **(H)** Histomorphological changes in the nasal mucosa revealed by Periodic Acid-Schiff (PAS) staining in different groups. **(I)** The goblet cell count in nasal mucosa of mice between groups (scale bar = 50 μm). **(J)** Statistical comparison of latency to locate buried food pellets via olfaction between groups, assessed using the buried food pellet test (BFPT). **(K)** Statistical comparison of latency to locate buried food pellets by visual assistance between groups, assessed using the BFPT. Bar values = mean ± standard error of the mean (SEM); n = 3 animals/group for histopathological staining, n = 12 animals/group for behavioral assessment and ELISA. All graphs were analyzed using t-test. *P < 0.05, **P < 0.01 and ***P < 0.001.

An OVA-induced AR mouse model was constructed over 12 consecutive weeks (**Figure 1A**). Behavioral assessment indicated that the frequency of nasal scratching within 3 min was markedly increased in the AR group compared with the control group (**Figure 1B**). As IgE in peripheral circulation constitutes a primary diagnostic criterion for AR, peripheral blood samples were collected and subjected to appropriate assays. Quantitative ELISA indicated that serum levels of IgE, IL-4, and IL-5 were substantially elevated in the AR group compared with that in the control group (**Figures 1C–E**). Microscopic evaluation of nasal mucosal sections stained with H&E showed enhanced eosinophil infiltration in the AR group (**Figure 1F**). Quantitative analysis confirmed that the mean number of eosinophils per field was significantly higher in the AR group compared with the control group (**Figure 1G**). PAS staining revealed goblet cell hyperplasia in the epithelium of the AR group (**Figure 1H**), and quantitative analysis showed a significant increase in the mean number of goblet cells per field in the AR group compared with the control group (**Figure 1I**). These data collectively verified the successful establishment of the mouse model of OVA-induced AR. The BFPT revealed that, compared with control mice, AR mice exhibited a prolonged latency in locating food pellets based on olfactory cues (**Figure 1J**). However, no significant differences were noted in the time taken to locate food with visual assistance (**Figure 1K**), indicating that OVA-induced AR mice exhibited quantifiable impairments in olfactory function.

### 3.2 Elevated CCL7 expression in the OB of AR-associated OD mice correlates with altered olfactory function

**Figure 2:**
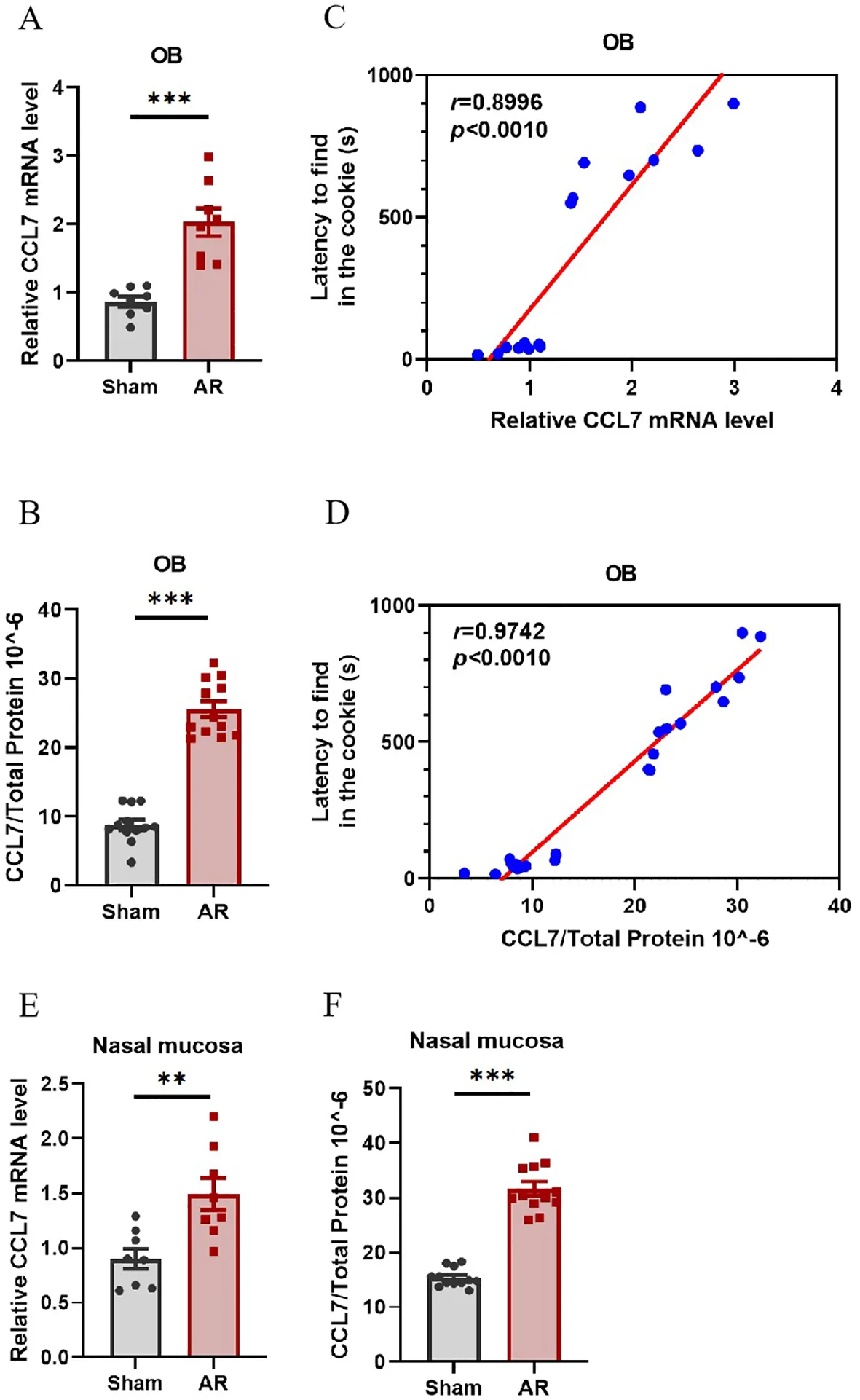
Changes in C-C motif chemokine ligand 7 (CCL7) levels in different groups, and the correlation with olfactory dysfunction (OD). **(A)** Statistical comparison of chemokine CCL7 in olfactory bulb (OB) of different groups, assessed using quantitative real-time polymerase chain reaction (qPCR). **(B)** Statistical comparison of CCL7 in OB of different groups, assessed using enzyme-linked immunosorbent assay (ELISA). **(C)** Correlation of CCL7 mRNA expression in OB with latency in buried food pellet test (BFPT). **(D)** Correlation of CCL7 protein level in OB with latency in BFPT. **(E)** Statistical comparison of chemokine CCL7 in the nasal mucosa of different groups, assessed using qPCR. **(F)** Statistical comparison of chemokine CCL7 in the nasal mucosa of different groups, assessed using ELISA. Bar values = mean ± standard error of the mean (SEM); n = 8 animals/group for qPCR, n = 12 animals/group for ELISA, n = 8 animals/group for correlation analysis using qPCR, n = 12 animals/group for correlation analysis using ELISA. All graphs were analyzed using t-test. *P < 0.05, **P < 0.01, ***P < 0.001.

Our previous study demonstrated that AR-associated OD is associated with microglia-mediated neuroinflammation in the OB (27). To further verify whether AR-associated OD is related to chemokine expression in the OB, we first assessed the expression of chemokines previously reported in the literature in both groups using qPCR. Except for elevated CCL7 levels (**Figure 2A**), the chemokine expression patterns in the OB of AR-associated OD mice did not differ significantly from those of the control group (**Supplementary Figures 1A–L**). The upregulation of CCL7 protein levels in the OB was subsequently confirmed using quantitative ELISA analysis (**Figure 2B**). These findings indicate elevated CCL7 expression in the OB of AR mice with OD. Further correlation analysis demonstrated that CCL7 expression, assessed at both transcriptional and translational levels, exhibited a positive association with the latency period required for mice to locate buried food pellets (*r* = 0.8996, **Figure 2C** and *r* = 0.8437, **Figure 2D**, respectively). These data suggest that OD in AR mice may be associated with increased CCL7 expression in the OB. Consistent with these findings, complementary assessment of nasal mucosa revealed concordant CCL7 elevation, as substantiated by both qPCR and ELISA (**Figures 2E, F**).

### 3.3 CCR3 is the only CCL7 receptor upregulated in the OB of AR-associated OD mice, and its expression localized to the microglia

**Figure 3:**
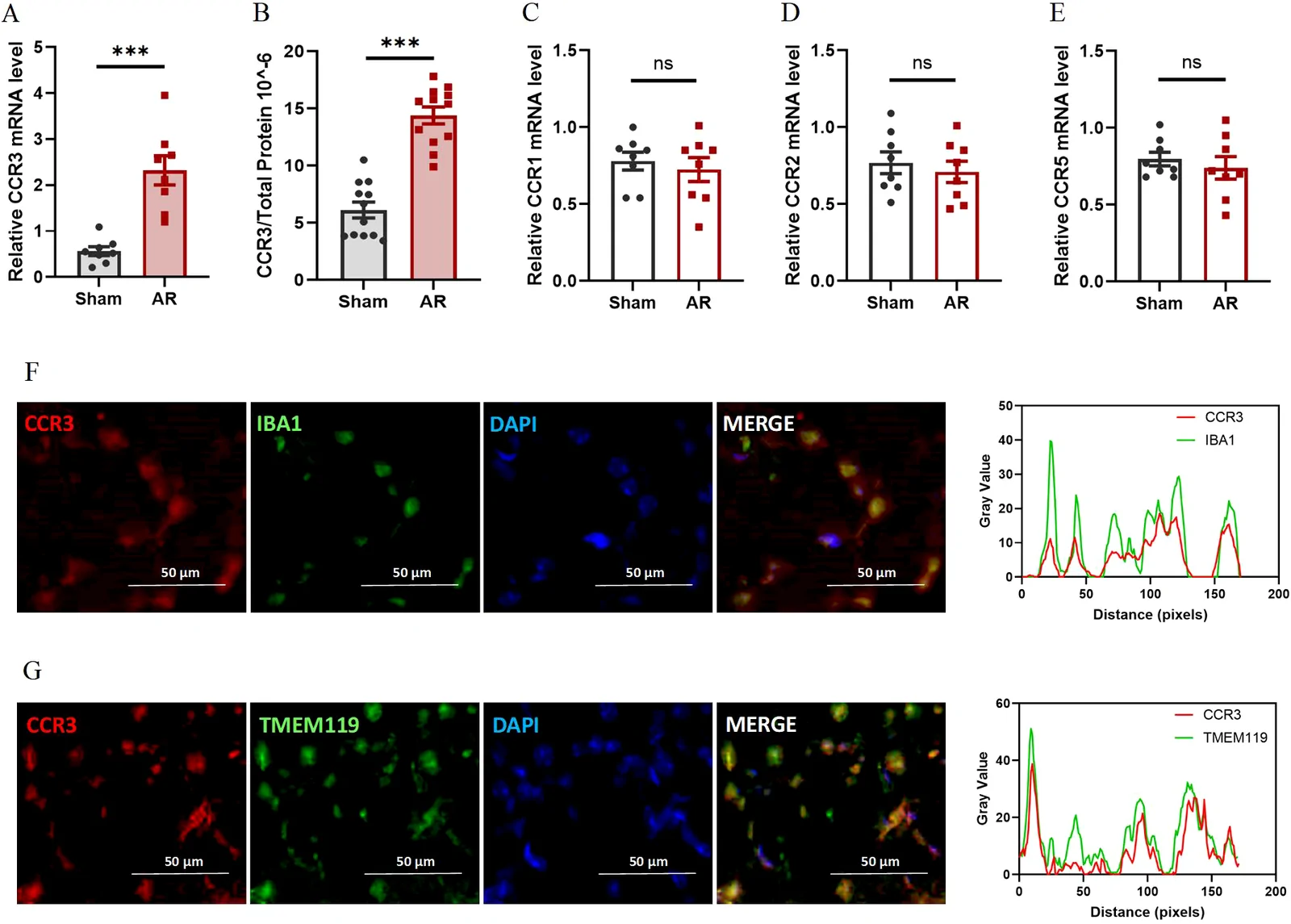
Changes in levels and localization of CCL7 receptors in olfactory bulb (OB) in different groups. **(A)** Statistical comparison of the CCL7 receptor CCR3 in OB of different groups, assessed using quantitative real-time polymerase chain reaction (qPCR). **(B)** Statistical comparison of the CCL7 receptors CCR3 in OB of different groups, assessed using enzyme-linked immunosorbent assay (ELISA). **(C–E)** Statistical comparison of the CCL7 receptors CCR1 **(C)**, CCR2 **(D)**, and CCR5 **(E)** in OB of different groups, assessed using qPCR. **(F)** Immunofluorescence staining revealed colocalization of CCR3 **(red)** with the microglial marker IBA-1 (green) in the OB, with the fluorescence intensity profile (right panel) displaying overlapping gray value peaks confirming their colocalization (scale bar = 50 μm). **(G)** Immunofluorescence staining revealed colocalization of CCR3 (red) with the microglial marker TMEM119 (green) in the OB, with the fluorescence intensity profile (right panel) displaying overlapping gray value peaks confirming their colocalization (scale bar = 50 μm). Bar values = mean ± standard error of the mean (SEM); n = 8 animals/group for qPCR, n = 12 animals/group for ELISA, n = 3 animals for immunofluorescence co-staining. All graphs were analyzed using t-test. *P < 0.05, **P < 0.01, ***P < 0.001, and ns, not significant.

CCL7 executes its diverse physiological and pathological functions primarily by binding to and activating specific chemokine receptors (30). The principal receptors identified for CCL7 include CCR1, CCR2, CCR3, and CCR5, each exhibiting distinct expression patterns and downstream signaling characteristics across different tissue types and pathological conditions (31). These receptor subtypes are differentially expressed across immune and neuronal cell populations, and the receptor-ligand interactions initiate complex intracellular signaling cascades, which regulate immune cell recruitment, inflammatory responses, and neuronal function (31). Given the critical importance of receptor availability and expression patterns in determining CCL7 functional outcomes, and considering the potential involvement of neuroinflammatory mechanisms in the context of AR-associated OD, dysregulation of the CCL7-receptor axis may represent a critical pathogenic mechanism underlying impaired olfactory performance. Therefore, we performed qPCR analysis to systematically assess the expression profiles of CCR1, CCR2, CCR3, and CCR5 in the OB tissue isolated from mice with AR-associated OD. The results revealed CCR3 as the sole receptor exhibiting significant differential expression, with substantially increased mRNA levels in the AR-associated OD group compared with controls (**Figure 3A**). The expression patterns of CCR1, CCR2, and CCR5 showed no significant intergroup variation (**Figures 3C–E**). Consistent with the transcriptional data, ELISA demonstrated increased CCR3 protein levels in the AR-associated OD group (**Figure 3B**). Previous studies from our laboratory established that activation of microglia in the OB constitutes an important mechanistic contributor to AR-associated OD (27, 28). To investigate the specific involvement of CCR3 in AR-induced neuroinflammatory processes underlying OD, we used immunofluorescence staining to determine the expression profile and cellular localization of CCR3 within OB microglia. We co-stained for CCR3 with the established microglial markers IBA1 and TMEM119. Immunofluorescence results verified the expression and specific localization of the CCL7 receptor CCR3 to microglia in the murine OB (**Figures 3F, G**). Fluorescence intensity profile analysis revealed that CCR3 fluorescence intensity (gray value) spatially overlapped with both IBA-1 and TMEM119, confirming their co-localization.

### 3.4 CCL7-CCR3 is an important mediator of OD and OB neuroinflammation in AR-associated OD mice

**Figure 4:**
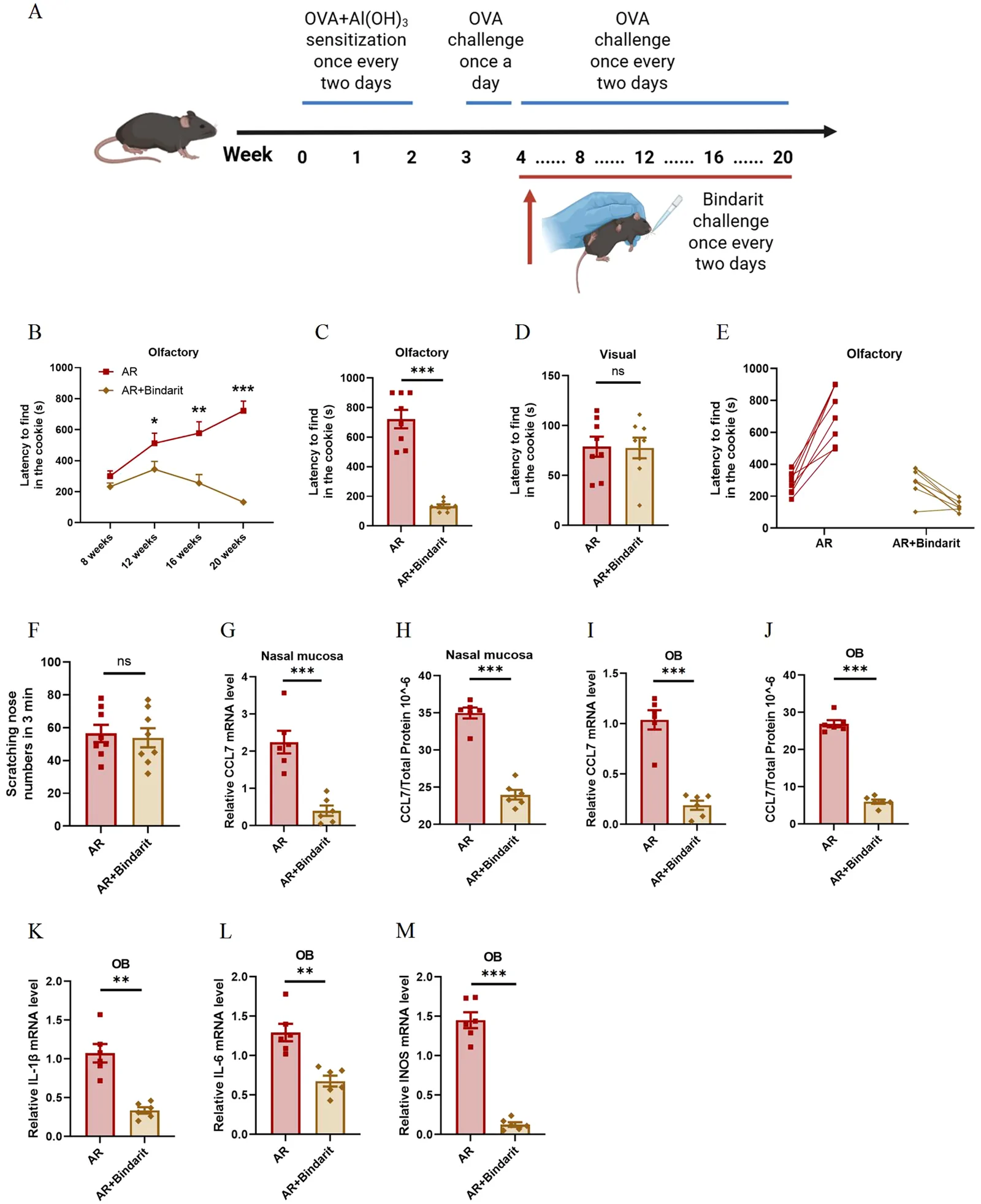
Changes in olfactory function and the expression of CCL7, IL-1β, IL-6, and iNOS following CCL7 inhibition in different groups. **(A)** Schematic illustration of the experimental protocol and temporal schedule for intranasal CCL7 inhibition (Bindarit) in AR mice. **(B)** Trend chart showing olfactory changes in different groups of mice during the treatment intervention period. **(C)** Statistical comparison of olfactory latency in locating buried food pellets via olfaction between different groups using the buried food pellet test (BFPT). **(D)** Statistical comparison of olfactory latency in locating buried food pellets under visual assistance between different groups using the BFPT. **(E)** Individual olfactory performance trajectories of each mouse in different groups commencing from the maintenance phase of AR model establishment to the end of the experiment. **(F)** Statistical comparison of nasal scratching frequency in 3 min among different groups. **(G)** Statistical comparison of chemokine CCL7 expression in the nasal mucosa of different groups assessed using quantitative real-time polymerase chain reaction (qPCR). **(H)** Statistical comparison of chemokine CCL7 expression in the nasal mucosa of different groups assessed using enzyme-linked immunosorbent assay (ELISA). **(I)** Statistical comparison of chemokine CCL7 expression in olfactory bulb (OB) of different groups assessed using qPCR. **(J)** Statistical comparison of chemokine CCL7 expression in OB of different groups assessed using ELISA. **(K)** Statistical comparison of changes in levels of proinflammatory cytokine IL-1β in OB of different groups assessed using qPCR. **(L)** Statistical comparison of changes in levels of proinflammatory cytokine IL-6 in OB of different groups assessed using qPCR. **(M)** Statistical comparison of changes in levels of proinflammatory cytokine iNOS in OB of different groups assessed using qPCR. Bar values = mean ± standard error of the mean (SEM); n = 8 animals/group for behavioral assessment, n = 6 animals/group for qPCR and ELISA. All graphs were analyzed using the t-test. *P < 0.05, **P < 0.01, ***P < 0.001, and ns, not significant.

**Figure 5:**
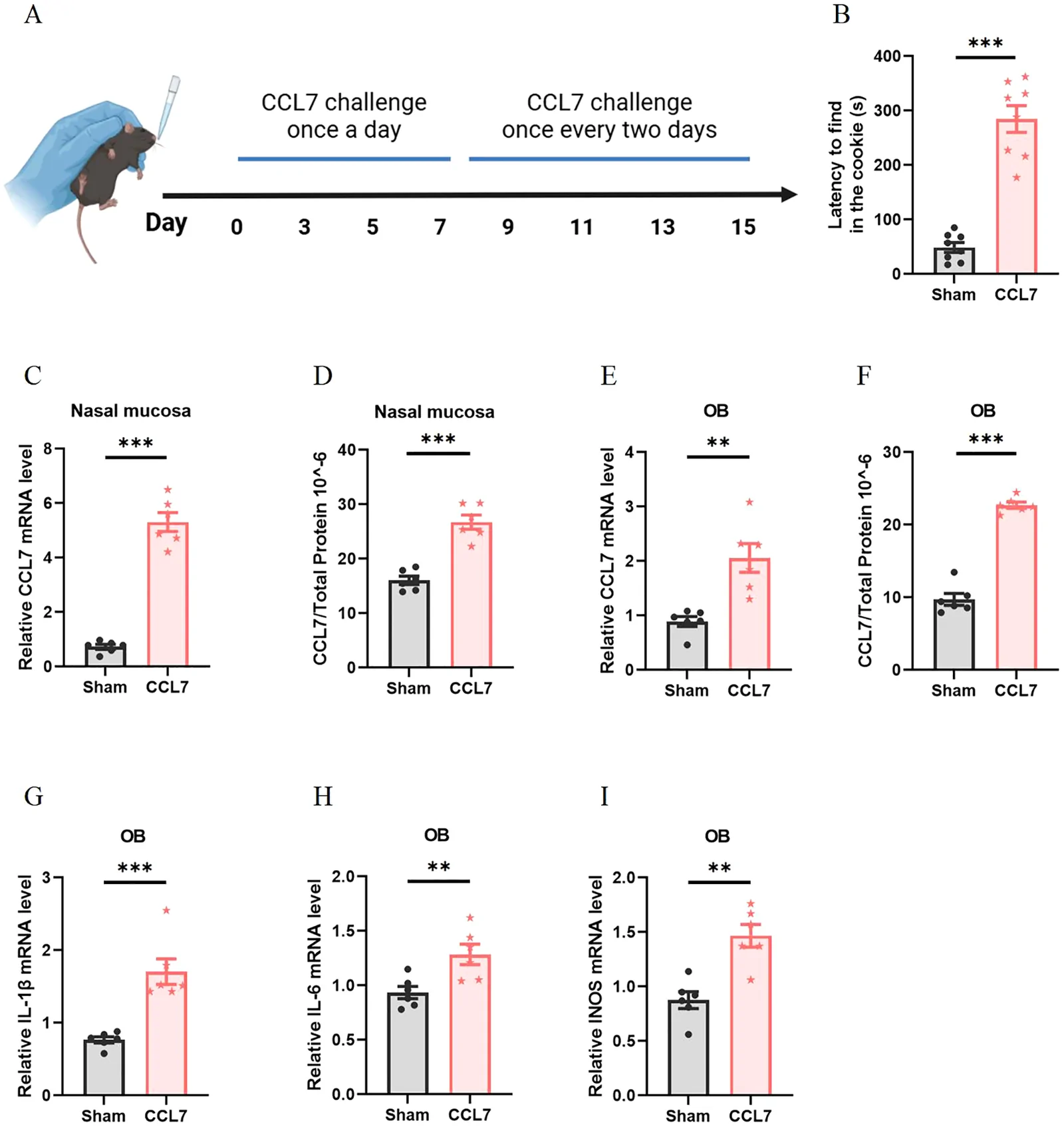
Changes in olfactory function and the expression of CCL7, IL-1β, IL-6, and iNOS following exogenous CCL7 administration in different groups. **(A)** Schematic illustration of the experimental protocol and temporal schedule for intranasal CCL7 administration in naive mice. **(B)** Statistical comparison of olfactory latency in locating buried food pellets via olfaction between different groups using the buried food pellet test (BFPT). **(C)** Statistical comparison of chemokine CCL7 expression in the nasal mucosa of different groups assessed using quantitative real-time polymerase chain reaction (qPCR). **(D)** Statistical comparison of chemokine CCL7 expression in the nasal mucosa of different groups assessed using enzyme-linked immunosorbent assay (ELISA). **(E)** Statistical comparison of chemokine CCL7 expression in olfactory bulb **(OB)** of different groups assessed using qPCR. **(F)** Statistical comparison of chemokine CCL7 expression in OB of different groups assessed using ELISA. **(G)** Statistical comparison of changes in levels of proinflammatory cytokine IL-1β in OB of different groups assessed using qPCR. **(H)** Statistical comparison of changes in levels of proinflammatory cytokine IL-6 in OB of different groups assessed using qPCR. **(I)** Statistical comparison of changes in levels of proinflammatory cytokine iNOS in OB of different groups assessed using qPCR. Bar values = mean ± standard error of the mean (SEM); n = 8 animals/group for behavioral assessment, n = 6 animals/group for qPCR and ELISA. All graphs were analyzed using the t-test. **P < 0.01, and ***P < 0.001.

To validate the involvement of CCL7 in AR-associated OD *in vivo*, we administered the CCL7 inhibitor Bindarit intranasally every other day, commencing at the maintenance phase of AR model establishment (**Figure 4A**). BFPT assessment demonstrated progressive, time-dependent restoration of olfactory function, with significant improvement achieved after 8 weeks of therapeutic intervention (corresponding to week 12 of the experimental timeline) and continuing thereafter (**Figure 4B**). At the terminal assessment point (corresponding to week 20 of the experimental timeline), Bindarit-treated mice exhibited markedly reduced latency in olfactory-based food detection compared with AR mice (**Figure 4C**), with no intergroup difference under visually-assisted conditions (**Figure 4D**). Individual olfactory performance trajectories are presented in **Figure 4E**. However, there was no significant difference in the frequency of nasal scratching between the two groups (**Figure 4F**). Both qPCR and ELISA consistently revealed significant downregulation of CCL7 expression in the nasal mucosa and OB of the Bindarit-treated group (**Figures 4G–J**). This was accompanied by reduced expression of the microglial neuroinflammatory markers IL-1β, IL-6, and inducible nitric oxide synthase (iNOS) in the OB (**Figures 4K–M**). To further validate the causative role of CCL7 in AR-associated OD, we assessed the effects of exogenous CCL7 administration in naive mice. Intranasal administration of CCL7 markedly impaired olfactory function and significantly upregulated the expression of microglial neuroinflammatory markers (IL-1β, IL-6, and iNOS) in the OB (**Figures 5A–I**). To directly demonstrate the specificity of the CCL7-CCR3 axis, we stereotaxically injected the CCR3-specific antagonist SB-328437 into the OB of AR mice with established OD, which significantly improved olfactory function and reduced OB protein levels of CCR3 (**Supplementary Figures 2A–C**). Collectively, these findings establish that the CCL7-CCR3 axis plays an important role in mediating OB microglial neuroinflammation and consequent OD in AR mice.

### 3.5 Epithelial-derived CCL7 contributes to CCR3-dependent microglial inflammatory activation *in vitro*

**Figure 6:**
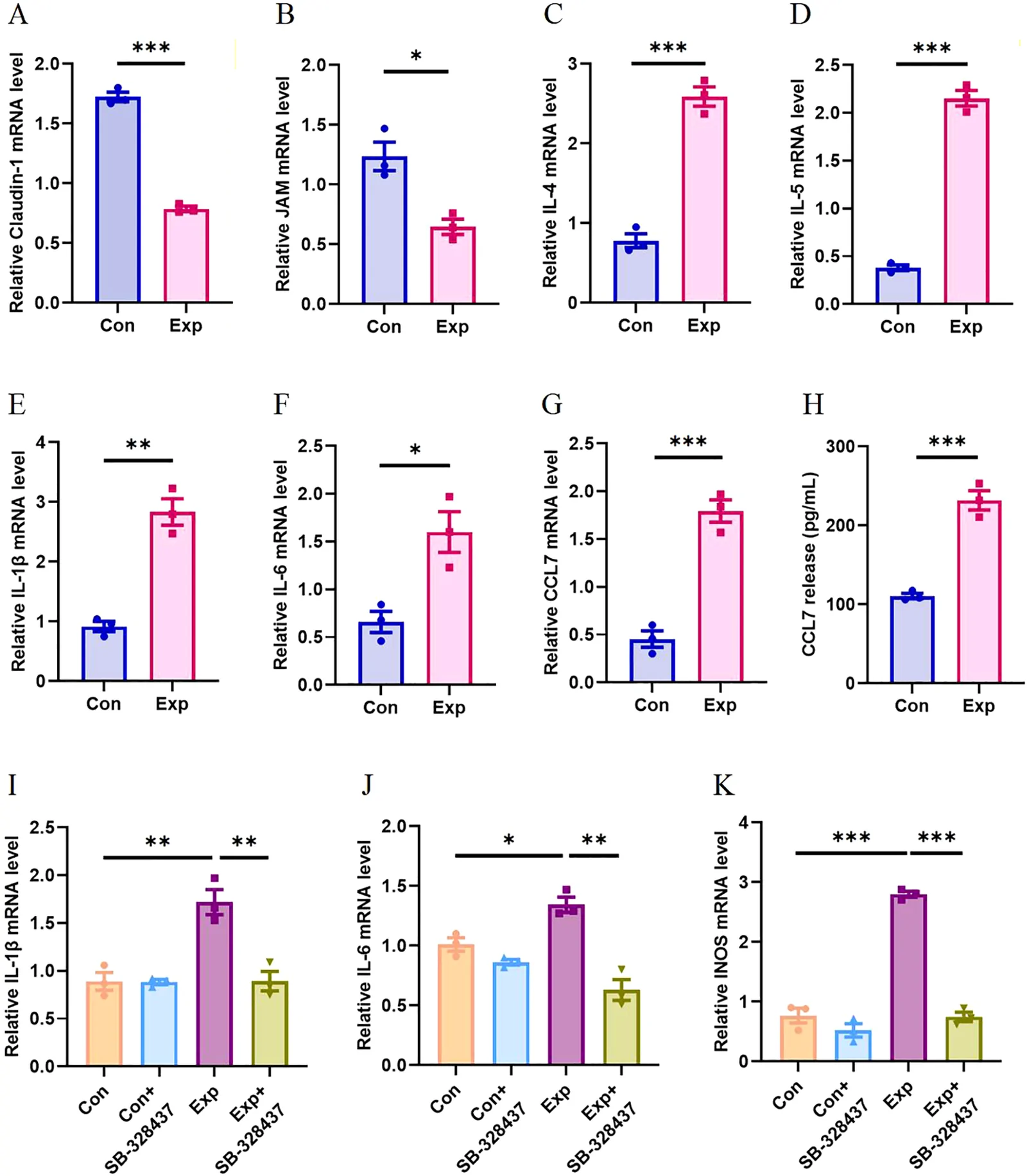
Effects of CCR3 inhibition on microglial cells during culture with conditioned medium (CM) from nasal mucosal epithelial cells following Der p 1 treatment. **(A)** Statistical comparison of Claudin-1 expression in human nasal epithelial cells (HNEpC) control and Der p 1-treated groups assessed using quantitative real-time polymerase chain reaction (qPCR). **(B)** Statistical comparison of JAM expression in HNEpC control and Der p 1 treatment groups assessed using qPCR. **(C)** Statistical comparison of IL-4 expression in HNEpC control and Der p 1 treatment groups assessed using qPCR. **(D)** Statistical comparison of IL-5 expression in HNEpC control and Der p 1 treatment groups assessed using qPCR. **(E)** Statistical comparison of IL-1β expression in HNEpC control and Der p 1 treatment groups assessed using qPCR. **(F)** Statistical comparison of IL-6 expression in HNEpC control and Der p 1 treatment groups assessed using qPCR. **(G)** Statistical comparison of CCL7 expression in HNEpC control and Der p 1 treatment groups assessed using qPCR. **(H)** Statistical comparison of CCL7 levels in CM between HNEpC control and Der p 1 treatment groups assessed using enzyme-linked immunosorbent assay (ELISA). **(I)** Statistical comparison of changes in expression of proinflammatory cytokine IL-1β in human microglial clone 3 (HMC3) cells of different groups assessed using qPCR. **(J)** Statistical comparison of the changes in expression of proinflammatory cytokine IL-6 in HMC3 of different groups assessed using qPCR. **(K)** Statistical comparison of the changes in expression of proinflammatory cytokine iNOS in HMC3 of different groups assessed using qPCR. Bar values = mean ± standard error of the mean (SEM); n = 3/group for qPCR and ELISA. The graphs corresponding to qPCR data were analyzed using t-test (two groups) or one-way ANOVA (four groups). The graph corresponding to ELISA data was analyzed using t-test. *P < 0.05, **P < 0.01 and ***P < 0.001.

Having established the involvement of the CCL7-CCR3 axis *in vivo*, we next sought to elucidate the underlying cellular mechanism using *in vitro* approaches. Given the inherent complexity of the *in vivo* environment and potential confounding variables, we established streamlined *in vitro* culture conditions to simulate the *in vivo* microenvironment, with the specific objective of elucidating the involvement of the CCL7-CCR3 axis in mediating OB microglial activation induced by nasal allergic responses. The qPCR analysis revealed that Der p 1 stimulation of nasal mucosal epithelial cells resulted in reduced mRNA expression of barrier integrity markers including Claudin-1 and junctional adhesion molecule (JAM) (**Figures 6A, B**), concomitant with upregulated expression of canonical AR inflammatory mediators, including IL-4, IL-5, IL-1β, and IL-6 (**Figures 6C–F**). Additionally, the experimental group exhibited elevated CCL7 mRNA expression compared with the control group (**Figure 6G**). Moreover, supernatant was collected as CM from Der p 1-stimulated nasal mucosal epithelial cells, and subsequently used in microglial co-culture experiments. ELISA confirmed markedly enhanced levels of CCL7 protein in the supernatant of the AR cellular model (**Figure 6H**). Following culture with this CM, HMC3 cells exhibited significantly increased mRNA expression of the neuroinflammatory markers IL-1β, IL-6, and iNOS relative to control conditions. Notably, pretreatment with SB-328437, a specific CCR3 inhibitor, prior to CM substantially attenuated the expression of these neuroinflammatory markers (**Figures 6I–K**). These findings suggest that CCR3 serves as an important mediator through which allergen-stimulated CCL7 release from nasal mucosal epithelium engages CCR3 to promote microglial activation and propagate neuroinflammatory responses.

## 4 Discussion

In the present study, we focused on identifying the mechanistic link between AR-associated OD and OB neuroinflammation. We demonstrated that CCL7 is significantly upregulated in the OB of AR mice with OD, with its receptor CCR3 selectively expressed and localized to activated microglia. Integrated *in vitro* and pharmacological evidence supports the involvement of the CCL7-CCR3 axis in microglial neuroinflammation and consequent OD. Collectively, these findings identify the CCL7-CCR3 axis as a novel and promising therapeutic target for AR-associated OD.

The prevalence of OD in AR has been increasingly recognized as a major clinical challenge. Epidemiological studies indicate that OD affects a substantial proportion of patients with AR, with the condition further exacerbating AR pathophysiology to establish a vicious cycle compromising patient wellbeing. Beyond impaired hedonic experience, olfactory loss poses safety risks by compromising the detection of environmental hazards including smoke, spoiled food, and toxic gases (32). Affected individuals frequently develop psychological comorbidities including anxiety and depression, amplifying the socioeconomic burden across individual, familial, and healthcare system levels (3, 4). However, the underlying mechanisms remain incompletely elucidated. Conventional explanations have emphasized mechanical nasal obstruction (33); however, the dissociation between nasal airflow and olfactory acuity (34), coupled with the ineffectiveness of decongestants versus the efficacy of anti-inflammatory interventions (35), compellingly indicate that immune-inflammatory mechanisms intrinsic to AR pathophysiology constitute the principal drivers of OD development. Recent investigations have demonstrated that allergic airway inflammation induces microglial activation, upregulation of proinflammatory cytokines (including tumor necrosis factor [TNF]-ɑ, IL-1β, and IL-6), and neuronal loss within the OB (27, 28, 36). These pathological alterations occur independently of mechanical obstruction and may represent a critical pathway through which AR engenders persistent or irreversible olfactory impairment. Microglia, as the resident immune cells of the central nervous system, serve as the principal responders to inflammatory challenge within the OB (37). Our laboratory previously established that OB microglial activation represents a pivotal mechanism underlying AR-associated OD, specifically identifying IL-6- and P2X7 receptor (P2X7R)-mediated signaling pathways as important drivers of this neuroinflammatory process (27, 28). These observations compel a shift in research focus toward elucidating the specific inflammatory mediators and signaling pathways, with the ultimate goal of identifying actionable therapeutic targets.

CCL7 (MCP-3), a versatile CC chemokine with pleiotropic binding to CCR1, CCR2, and CCR3, has been established as a key participant in AR and a critical driver of microglial neuroinflammation (23, 24). Beyond peripheral allergic inflammation, CCL7 has been implicated in diverse neurological conditions including multiple sclerosis (38), Alzheimer’s disease (39), and COVID-19-associated neurological manifestations (40). Building upon this evidence, we investigated the potential involvement of CCL7 in AR-associated OD and demonstrated that CCL7-CCR3 signaling mediates microglial activation within the OB, establishing a “nose-to-brain” inflammatory signaling pathway. Therapeutic inhibition of this axis through intranasal CCL7 inhibitor administration progressively improved olfactory function and attenuated neuroinflammatory markers. These results position CCL7-CCR3 as a novel and promising clinically actionable biomarker and therapeutic target for AR-associated OD. Regarding the pharmacological tools used, we acknowledge that Bindarit is not exclusively selective for CCL7; however, our conclusion is supported by convergent evidence from multiple approaches, including exogenous CCL7 administration in naive mice and CCR3-specific antagonism with SB-328437 in AR mice with established OD. Notably, Bindarit was administered therapeutically during the maintenance phase after AR had been established in this study. Although we observed marked improvements in olfactory function and OB neuroinflammation, nasal scratching showed no significant difference between groups. This dissociation suggests that CCL7-CCR3 inhibition attenuates neuroinflammation and restores olfactory function but may not directly relieve pruritus in established AR, possibly because behavioral symptoms are regulated by multiple pathways beyond CCL7, requiring earlier or combined intervention.

Nevertheless, the present study has several limitations. First, genetic validation is lacking. The pharmacological approach employed herein does not exclude contributions of other CCL7 receptors or compensatory mechanisms. Although we identified CCR3 as the principal mediator of the effects, definitive mechanistic verification requires OB microglia-specific CCR3 conditional knockout mice. However, owing to technical hurdles, no such models have been reported in the literature or are currently available as commercial strains. Although systemic knockouts exist, they introduce considerable confounding factors, rendering them unsuitable for precise mechanistic dissection. Second, direct *in vivo* tracing of epithelial-derived CCL7 was not performed. Although the *in vitro* and *in vivo* findings collectively support the involvement of a nose-to-brain inflammatory axis, we did not directly trace CCL7 from nasal epithelium to OB in living animals. Future studies employing labelled CCL7 or epithelial-specific reporter systems would provide more definitive evidence for this transduction pathway. Third, the CM approach does not exclude contributions from other inflammatory molecules. Although CCR3 antagonism supports CCL7 as the predominant effector, potential interplay with other mediators (including IL-1β, IL-6, and P2X7R identified in our previous work (27, 28)) warrants investigation. Fourth, clinical translational validation remains insufficient. Interspecies differences and the impracticality of human OB sampling limit the ability to perform direct validation. Although murine models provide valuable mechanistic insights, they do not fully recapitulate the complex heterogeneous clinical phenotypes of human AR. Whether minimally invasive specimens (nasal lavage fluid and peripheral blood) can serve as surrogate biomarkers requires urgent exploration.

While our findings provide foundational evidence for CCL7-CCR3 as a critical axis in AR-associated OD, deeper mechanistic insight awaits future investigation. Some priorities warrant further investigation. Further research is needed on downstream intracellular pathways related to CCL7 signaling, such as the NOD-like receptor protein 3 (NLRP3) inflammasome and IL-1β pathway associated with AR pathophysiology. Our previous work identified IL-1β, IL-6, and P2X7R as key drivers of OB neuroinflammation (27, 28); however, crosstalk with CCL7-CCR3 remains to be clarified. We fully acknowledge that AR-associated OD is a multifactorial disease. These mediators and CCL7 represent distinct yet complementary upstream initiating factors that converge on a common endpoint: microglial neuroinflammation in the OB. Whether CCL7-CCR3 operates sequentially, in parallel, or interdependently with these established mediators or pathways requires clarification to inform combinatorial therapeutic design. Additionally, whether an optimal intervention window exists remains unknown. Given the time-dependent improvement in olfactory function following CCL7 inhibition, an important question is whether early treatment can prevent irreversible damage, or whether later intervention can reverse established pathology. Longitudinal studies with varied treatment initiation timepoints are essential to guide clinical translation.

From a translational perspective, CCL7 or CCR3-targeted monoclonal antibodies represent a promising alternative to small-molecule inhibitors. Antibody-based therapeutics offer enhanced specificity, prolonged half-life, and reduced off-target effects. Intranasal delivery of such biologics may achieve localized OB modulation while minimizing systemic exposure (41). Future studies should prioritize preclinical development and pharmacokinetic evaluation of such targeted biologics for intranasal administration.

## 5 Conclusion

In conclusion, we identified the CCL7-CCR3 signaling axis as an important mechanism linking nasal allergic inflammation to OB neuroinflammation and OD. These findings advance our understanding of AR pathophysiology, identifying the CCL7-CCR3 axis as a novel therapeutic target. Given the substantial clinical burden of AR-associated OD and the inadequacy of current treatments, targeted modulation of this axis offers a potential therapeutic avenue for AR-associated OD and holds considerable translational promise. Future directions include defining optimal intervention windows, determining disease-modifying versus symptomatic efficacy, and validating clinical applicability through biomarker studies.

## Data Availability

The original contributions presented in the study are included in the article/**Supplementary Material**. Further inquiries can be directed to the corresponding authors.

## Supplementary material

Supplementary Figure 1 Changes in chemokine expression in olfactory bulb (OB) in different groups. **(A)** Statistical comparison of chemokine CCL2 expression in OB of different groups assessed using quantitative real-time polymerase chain reaction (qPCR). **(B)** Statistical comparison of chemokine CCL3 expression in OB of different groups assessed using qPCR. **(C)** Statistical comparison of chemokine CCL5 expression in OB of different groups assessed using qPCR. **(D)** Statistical comparison of chemokine CCL11 expression in OB of different groups assessed using qPCR. **(E)** Statistical comparison of chemokine CCL12 expression in OB of different groups assessed using qPCR. **(F)** Statistical comparison of chemokine CCL20 expression in OB of different groups assessed using qPCR. **(G)** Statistical comparison of chemokine CCL24 expression in OB of different groups assessed using qPCR. **(H)** Statistical comparison of chemokine CCL26 expression in OB of different groups assessed using qPCR. **(I)** Statistical comparison of chemokine CXCL2 expression in OB of different groups assessed using qPCR. **(J)** Statistical comparison of chemokine CXCL9 expression in OB of different groups assessed using qPCR. **(K)** Statistical comparison of chemokine CXCL10 expression in OB of different groups assessed using qPCR. **(L)** Statistical comparison of chemokine CXCL16 expression in OB of different groups assessed using qPCR. Bar values = mean ± standard error of the mean (SEM); n = 8 animals/group for qPCR. All graphs were analysed using the t-test. ns, not significant.

Supplementary Figure 2 Changes in olfactory function and the expression of CCR3 following CCR3-specific antagonist in different groups. **(A)** Statistical comparison of olfactory latency in locating buried food pellets via olfaction between allergic rhinitis (AR) and AR mice treated with CCR3-specific antagonist SB-328437 (AR+SB-328437) group using the buried food pellet test (BFPT). **(B)** Individual olfactory performance trajectories of each mouse in AR and AR+SB-328437 group. **(C)** Statistical comparison of CCR3 expression in the olfactory bulb (OB) of AR and AR+SB-328437 group assessed using enzyme-linked immunosorbent assay (ELISA). Bar values = mean ± standard error of the mean (SEM); n = 6 animals/group for behavioral assessment and ELISA. All graphs were analysed using the t-test. ***P < 0.001.
